# Validation studies of the ParaDNA^®^ Intelligence System with artificial evidence items

**DOI:** 10.1080/20961790.2019.1665159

**Published:** 2019-12-06

**Authors:** Min Li, Ruiyang Tao, Wei Zhou, Yanan Li, Meng Meng, Yilun Zhang, Linsheng Yu, Liqin Chen, Yingnan Bian, Chengtao Li

**Affiliations:** a Institute of Forensic Medicine, West China School of Basic Medical Sciences & Forensic Medicine, Sichuan University, Chengdu, China; b Shanghai Key Laboratory of Forensic Medicine, Shanghai Forensic Service Platform, Academy of Forensic Sciences, Ministry of Justice, P.R. China, Shanghai, China;; c The Jackson Laboratory for Genomic Medicine, Farmington, CT, USA; d Department of Forensic Medical, School of Basic Medical Sciences, Inner Mongolia Medical University, Hohhot, China; e Department of Histology and Embryology, Harbin Medical University, Harbin, China; f School of Basic Medicine, Baotou Medical College, Baotou, China; g Department of Forensic Medicine, School of Basic Medical Science, Wenzhou Medical University, Wenzhou, China; h School of Basic Medicine, Inner Mongolia Medical University, Hohhot, China; i Zhejiang Provincial Key Laboratory of Medical Genetics, Key Laboratory of Laboratory Medicine, Ministry of Education, China, School of Laboratory Medicine and Life Sciences, Wenzhou Medical University, Wenzhou, Zhejiang, China

**Keywords:** Forensic sciences, forensic genetics, ParaDNA^®^ Test Intelligence System, rapid DNA, melt curve analysis, HyBeacon^TM^ probes

## Abstract

Short tandem repeat (STR) profiling is one of the mostly used systems for forensic applications. In certain circumstances, STR profiling is time-consuming and costly, which potentially leads to delays in criminal investigations. LGC (Laboratory of the Government Chemist, UK) Forensics has developed a robust STR profiling platform called the ParaDNA^®^ Intelligence Test System which can provide early tactical intelligence and aid investigators in making informed decisions on sample prioritization for detection. Here, we validated the ParaDNA intelligence test for its application in forensic cases using a range of mock evidence items following guidelines set by the Scientific Working Group on DNA Analysis Methods (SWGDAM). Specifically, we tested the sensitivity and accuracy of the ParaDNA intelligence test, as well as the success rates for detecting mock samples and for use in case scenarios. Our findings demonstrate that the ParaDNA intelligence test generates useful DNA profiles, especially for samples such as blood, saliva, and semen that contain ample DNA, indicating the benefits of including ParaDNA as a prior step in forensic STR profiling pipelines.

## Introduction

In forensic genetics, short tandem repeat (STR) profiling is a primary scientific tool widely used in human identification and paternity testing, together with other applications [1]. Accordingly, many commercial forensic kits are presently available for STR profiling. Generally, in a forensic laboratory, DNA is extracted from biological materials; it is then purified and quantified before being amplified using polymerase chain reaction (PCR) and separated according to different product lengths caused by different repeat numbers of the motif using capillary electrophoresis (CE) technology. These steps, coupled with interpretation of the results, need trained practitioners and are time-consuming when screening a large number of evidence items [2].

To overcome this limitation, a number of methods have been proposed, such as direct PCR [3], rapid DNA technologies [4] and automated sample handling [5]. However, it is expensive to use these methods and requires substantial expertise for both operation and interpretation of results.

Accordingly, LGC (Laboratory of the Government Chemist, UK) Forensics has developed a ParaDNA^®^ Intelligence Test System to enable rapid STR profiling with minimal expertise. The system can analyze samples at five STR loci (D3S1358, D16S539, D8S1179, D18S51 and TH01) that are included in the Combined DNA Index System (CODIS), as well as amelogenin (the gender typing locus). The ParaDNA intelligence test can process samples without DNA extraction or purification, and the results can be generated in approximately 75 min [3]. The results generated from the ParaDNA^®^ Intelligence Test System are expected to be used to triage samples, indicating a subset of samples that should be further fully profiled. In addition, the ParaDNA intelligence test can provide immediate tactical intelligence to aid investigators by rapidly filtering potential suspects, linking scenes, and searching specified databases for potential suspects [6].

Despite its great potential, the ParaDNA intelligence test has not been validated in forensic science laboratories in China, or at least, the results of such validations are not reported. Here, we systematically validated the ParaDNA intelligence test using a range of mock evidence items following guidelines set by the Scientific Working Group on DNA Analysis Methods (SWGDAM) [7]. We tested the sensitivity and accuracy of the ParaDNA intelligence test, as well as the success rates for detecting mock samples and for use in case scenarios. Other areas such as reproducibility, sample inhibition/degradation and sample removal have been tested extensively by other laboratories [6]. Our findings demonstrate that the ParaDNA^®^ Intelligence System generates useful DNA profiles for samples with abundant DNA, such as blood, saliva and semen. The results suggest that implementing the ParaDNA intelligence test in standard forensic analyses pipelines could enable early decision-making and consequently result in more efficient forensic investigation.

## Materials, methods and techniques

### ParaDNA^®^ Intelligence System

The ParaDNA^®^ Intelligence System is composed of the ParaDNA screening unit, the ParaDNA sample collector and the ParaDNA intelligence test kit [6, 8], which co-amplifies D3S1358, D16S539, D8S1179, D18S51 and TH01 STR loci and the amelogenin marker. Firstly, biological samples and evidence items are transferred with the ParaDNA sample collector into four reaction plates that are included in the test kit. Afterwards, the reaction plates are placed onto the instrument to conduct PCR and melt curve analyses using fluorescent HyBeacons^®^ technology, which takes approximately 75 min. Finally, STR profiles are called automatically and are displayed by the ParaDNA software, which also provides profile searching and comparison functions.

The ParaDNA software controls the instrument and displays the results. The results are delivered to the user as recorded profile calls for each sample without displaying the underlying DNA melt data. However, samples with low level DNA (such as touch DNA) are more vulnerable to miscalls due to stochasticity and produce less than seven alleles across the six markers. Therefore, the ParaDNA software displays “Insufficient DNA to determine a profile” when any profiles are detected with fewer than seven alleles. In addition, another software, named ParaDNA Data Analysis Software, provided by LGC, is used when we expected to obtain a more complete profile, as sometimes the ParaDNA profiles are not complete when samples with low levels of DNA are tested. Moreover, the ParaDNA Data Analysis Software is also used when the ParaDNA software displays insufficient data or when the ParaDNA software indicates a mixture or possible mixture. The ParaDNA Data Analysis Software is designed for use by operators with experience of interpreting ParaDNA results. The ParaDNA Data Analysis results are those generated by the software, and have not been subjectively interpreted by the expert user so as not to introduce user’s bias. The “Results and discussion” section illustrates that the ParaDNA Data Analysis Software produces more useful allelic information than the ParaDNA software. So all the data produced are displayed by the two softwares (ParaDNA Software and ParaDNA Data Analysis Software).

Throughout the study, the results use “usable profiles” as a measurement of the ParaDNA intelligence test performance. The metric “usable profiles” is based on the ParaDNA software only displaying a profile when it detects seven or more alleles (≥7 allele calls). Blackman et al. [6] reported that these data can be used for intelligence or triage purposes. Simply speaking, when the ParaDNA software detects less than seven alleles, it displays “insufficient DNA to determine a profile”; the number of alleles is recognized as zero. After being re-checked by the ParaDNA Data Analysis Software, the actual number of alleles underlying the call of zero allele could be counted. This does not mean there is no DNA, but there is not enough DNA to get a confident profile. Complete profiles of 12 alleles are not always obtained when testing some high specification samples, such as touch DNA and so on. Software thresholds and guidelines focus more on accuracy than detection frequency to ensure that users have confidence in the results presented by the system.

### Samples

The ParaDNA intelligence test is designed to amplify human DNA that is sampled from an evidence item or swab. The types of samples that an end-user could encounter are diverse in their DNA contents. To account for this variability, mocked-up case samples were chosen including (i) blood on glass, on Flinders Technology Association (FTA) cards (Thomas Scientific, Swedesboro, NJ, USA) and on denim; (ii) saliva on glass, buccal swabs, drink bottles and smoked cigarettes; (iii) semen on glass and on carpet; and (iv) touch DNA on clothing and on a mobile phone. Among them, touch DNA samples are often a challenge in forensic science. All samples were collected from volunteers with written consent.

All samples (except buccal samples, smoked cigarettes, drink bottles and touch DNA) were prepared by pipetting biological materials onto the corresponding substrates and air-drying for a minimum of 2 h. Next, samples were collected from these items following LGC’s ParaDNA User Guide [9]. Most mock samples were collected directly from the items using the ParaDNA sample collector, with the exceptions of blood, saliva and semen on glass, which were first swabbed off the glass using a wet cotton swab and subsequently sampled using the ParaDNA sample collector. The sampling methods reflect the way that samples would typically be collected by a crime scene investigator or a forensic scientist in a live casework environment.

### Sensitivity study

In live casework scenarios, the levels of DNA submitted are uncontrollable which means it is important to determine the upper and lower limits with which the ParaDNA intelligence test will detect a DNA source. Blood, saliva, and semen are three of the most commonly encountered body fluids in forensic genetics. To evaluate the sensitivity of the ParaDNA intelligence test in recovering allele calls from these three common fluid types, we used ParaDNA software and ParaDNA Data Analysis Software to quantify the average number of generated allelic calls for the mock samples that were prepared according to the corresponding volumes (Table 1). Here neat samples were regarded as the raw liquid, dilution (1/10) samples were prepared by 5 µL neat samples with 45 µL ddH_2_O, and dilution (1/100) samples were prepared by 0.5 µL neat samples with 49.5 µL ddH_2_O.

**Table 1.** Samples prepared for the sensitivity study.

Volume	Blood on cotton swab (*N*=23)	Blood on FTA card (*N*=24)	Saliva on cotton swab (*N*=24)	Semen on cotton swab (*N*=29)
Neat 10 μL	*n* = 6	*n* = 6	*n* = 6	*n* = 6
Neat 5 μL + 45 μL ddH_2_O (dilution 1/10)	*n* = 6	*n* = 6	*n* = 6	*n* = 6
Neat 5 μL	–	–	–	*n* = 2
Neat 1 μL	*n* = 5	*n* = 6	*n* = 6	*n* = 9
Neat 0.5 μL + 49.5 μL ddH_2_O (dilution 1/100)	*n* = 6	*n* = 6	*n* = 6	*n* = 6

Neat: raw liquid; *n*: the number of replicates; –: not tested.

It is important to note that in the sensitivity study, the samples we used were biological samples that we simulated on live cases rather than DNA samples, so we could not directly quantify the upper and lower limits of DNA detection; thus, we used the volume of the sample to replace the amount of DNA as the quantitative index. Generally, the larger the volume, the higher the amount of DNA.

### Success rates of profiling call in different substrates

To identify the various samples and different substrate types that could be detected with the ParaDNA System, the profiling call success rates of the ParaDNA intelligence test was assessed by testing a series of mock samples prepared as described in “Samples” section. Among these samples, blood, saliva and semen used a neat 10 µL volume of raw liquid, dispensing on different substrates, and buccal samples, smoked cigarettes, drinks bottle and touch DNA were detected directly. The profile call success rates were measured by the percentage of “usable profiles” generated and the average number of alleles displayed.

### Application of the ParaDNA intelligence test in different mock case scenarios

To test whether the STR profile information provided by the ParaDNA intelligence test can reliably support the generation of rapid, cost effective, tactical intelligence in live investigations, we applied the ParaDNA intelligence test in two mock case scenarios that are regularly encountered in forensic caseworks. The ParaDNA^®^ Intelligence System has a “search and compare” function that can identify evidence items that potentially contain DNA from a specific subject or from a different evidence item. This function allows users to quickly link or exclude suspects to a scene and even to infer relatedness between crime scenes. In order to test the “search and compare” function, an unrelated reference buccal sample was used to replace the “true” suspect’s buccal sample in the first mock case scenario.

The first mock case scenario represented a sexual assault, with the victim claiming that she was assaulted in the room where the victim and the suspect had bled at the scene. Evidence for this scenario included blood stains on the clothing of the victim and the suspect, as well as a seminal stain in the victim’s bedroom. Additionally, buccal samples from the victim and the “unrelated suspect” were found at the scene.

The second case scenario represented a case of human trafficking, with the suspect and the victim found in a vehicle known to be used for human trafficking and parked outside a house. The suspect claimed that the victim was his child and that neither of them had ever been in the house. Evidence for the second scenario included blood stains, drinking containers, cigarette ends and shirts found in the house, as well as buccal samples collected from the victim and the suspect.

### Cross-validation

The accuracy of the ParaDNA intelligence test was assessed by comparing the STR profiles of all the above-mentioned samples that had undergone the ParaDNA intelligence test with that generated from corresponding reference buccal samples using the Goldeneye^TM^ DNA ID System 20 A Kit (Goldeneye, Co, Ltd, Beijing, China) following the manufacturer’s recommended protocol.

## Results and discussion

### Sensitivity study

**Figure 1.**
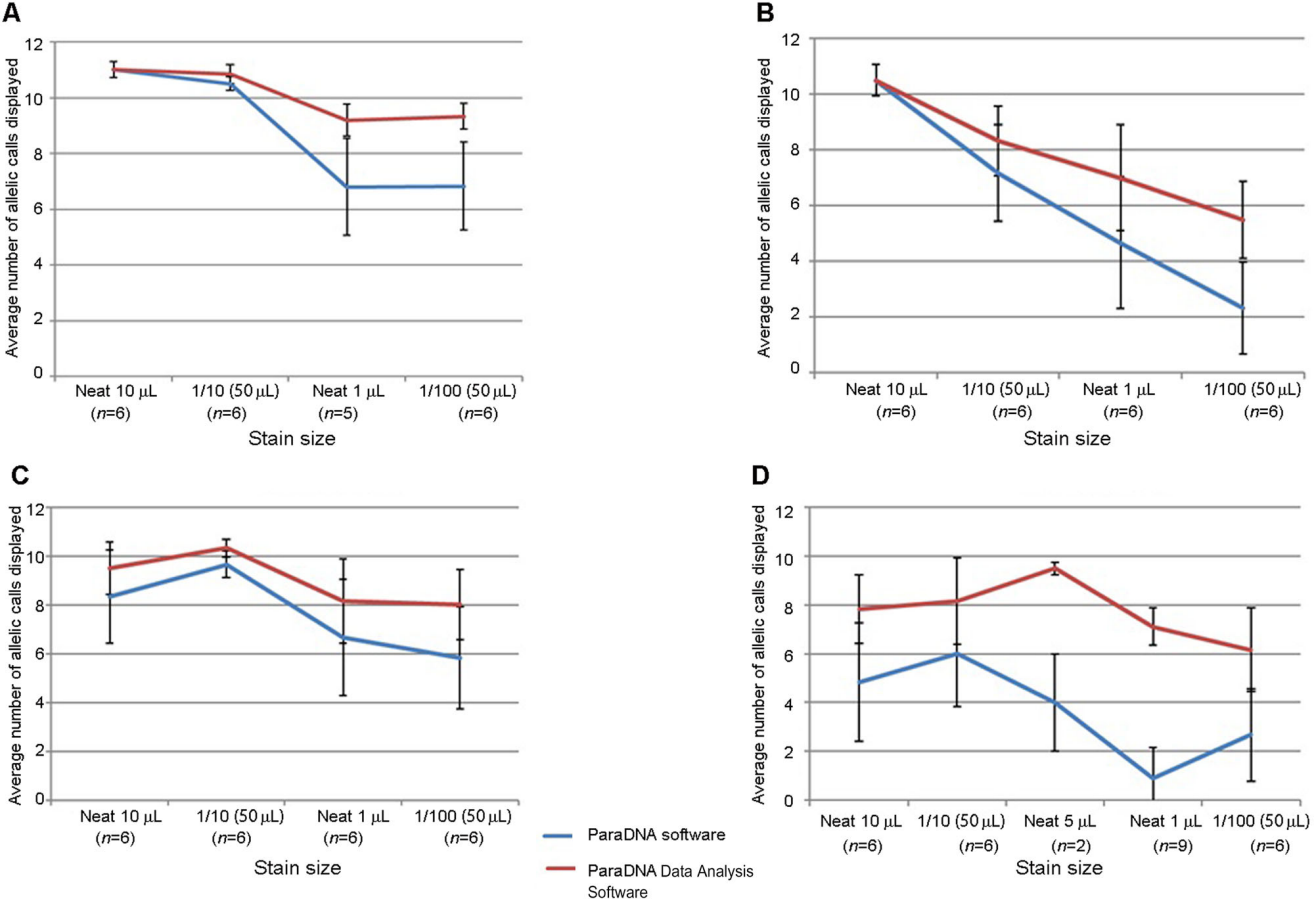
The average number of alleles identified using the ParaDNA intelligence test and analyzed by ParaDNA software and ParaDNA Data Analysis Software, respectively. Average number of allelic cells for blood (A), saliva (C), and semen (D) on swabs, as well as blood on FTA cards (B).

In the sensitivity study, we calculated the average number of alleles displayed by the ParaDNA software and the ParaDNA Data Analysis Software from a series of samples of blood, saliva and semen on swabs, as well as blood on FTA cards (Figure 1). “Usable profiles” (≥7 alleles) was used as a measurement. As mentioned above, we used the volume of samples to represent the amount of DNA. For blood on swabs, the blood amount was neat 1 µL that obtained the least number of alleles from the ParaDNA software and the ParaDNA Data Analysis Software (Figure 1A). For blood on FTA cards and saliva on swabs, the least number of obtained alleles was based on the amounts of blood and saliva that were both dilution 1/100 (50 µL) (Figure 1B and C). For semen on swabs, neat 1 µL semen produced the least number of alleles from the ParaDNA software, but when checking by ParaDNA Data Analysis Software, dilution 1/100 (50 µL) semen produced the least number of alleles (Figure 1D). By using the ParaDNA Data Analysis Software, the semen samples of neat 1 µL that were labeled as “insufficient DNA to determine a profile” by ParaDNA software displayed seven to ten alleles accurately. These results illustrated that the ParaDNA Data Analysis Software produced more useful allelic information than the ParaDNA software.

For different volumes of blood samples and saliva samples present on swabs, ≥7 alleles (usable profile) were identified by the ParaDNA Data Analysis Software using dilution 1/100 (50 µL) samples (Figure 1(A) and (C)). For the samples of blood on FTA cards and semen on swabs, an average of usable profile (≥7 allele call profiles) was returned by the ParaDNA Data Analysis Software using neat 1 µL samples (Figure 1(B) and (D)). In general, the blood samples and saliva samples present on swabs could be detected more sensitively than the blood on FTA cards and semen on swabs by the ParaDNA intelligence test with the minimum amounts as dilution 1/100 (50 µL) and neat 1 µL, which were all satisfactory for the forensic science application.

### Success rates of profiling calls on different substrates

**Figure 2.**
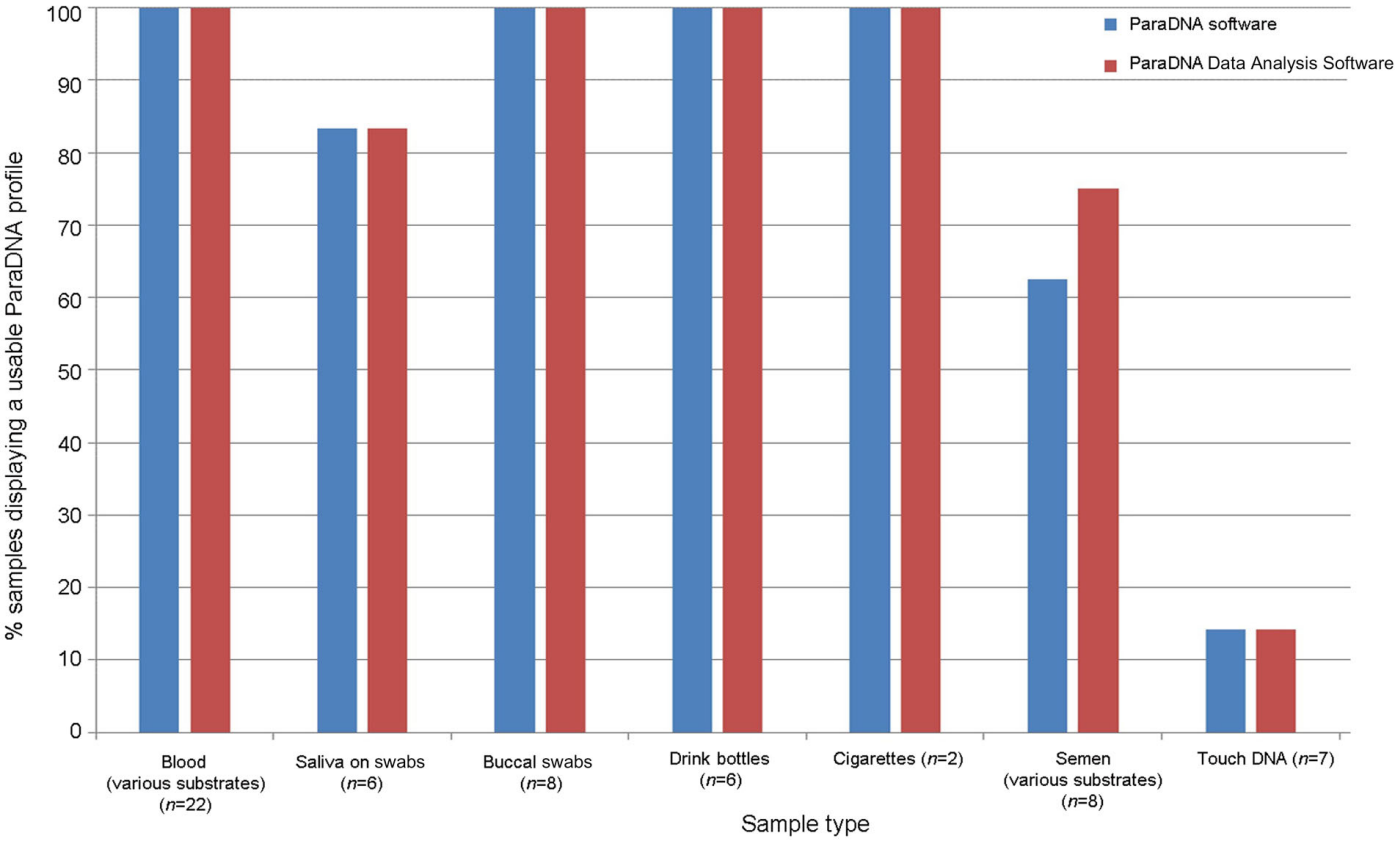
Percent of samples of different substrates displaying a usable profile (≥7 allele calls) with the ParaDNA intelligence test analyzed by the ParaDNA software and ParaDNA Data Analysis Software.

The success rates of profiling calls by the ParaDNA intelligence test was assessed using a range of sample types on different substrates. Being able to produce seven or more alleles (usable profiles) means that profiling is successful. Figure 2 shows the percent of samples displaying a usable ParaDNA profile for each sample type by using ParaDNA software and ParaDNA Data Analysis Software. On the whole, the results of the two softwares were consistent. The blood samples generated usable profiles in 100% of the tested samples. The saliva samples (buccal samples, drink bottles and cigarettes) also had a 100% success rate, while the success rate was more than 80% in the case of saliva on swabs. Usable profiles could be generated from more than 60% (above 75% checked by ParaDNA Data Analysis Software) of semen samples on different substrates. Items named touch DNA (cellular samples on clothing and mobile phone) yielded a low number of alleles, with only 14% of profiles being usable.

**Figure 3.**
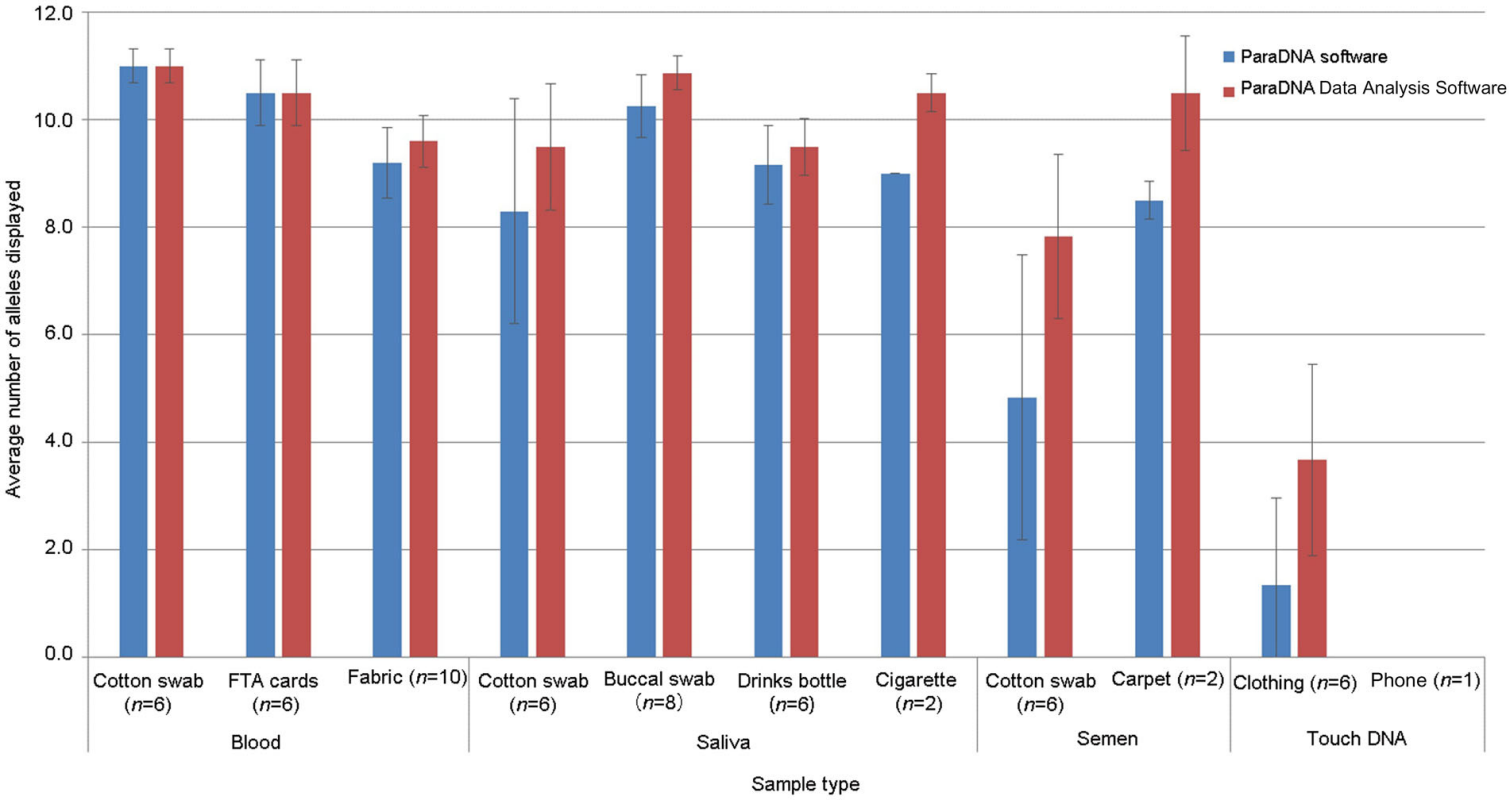
Average number of alleles identified for four types of samples on different substrates using the ParaDNA software and ParaDNA Data Analysis Software. Error bar represents the standard deviation.

Except for semen on swabs and touch DNA, other sample types could be detected and obtained usable profiles (≥7 alleles detected) using the ParaDNA intelligence test on different substrates (Figure 3) analyzed by ParaDNA software. Semen on swabs that was analyzed by ParaDNA Data Analysis Software yielded the average number of 7.6 alleles that could generate usable profiles, which illustrated the better performance of the ParaDNA Data Analysis Software again. The ParaDNA^®^ Intelligence System performed consistently well when detecting sample types that were expected to contain high amounts of DNA, such as blood, saliva and semen samples. The touch items gave poor results both analyzed by ParaDNA software and ParaDNA Data Analysis Software, as would be expected for those low-level DNA samples, such as cellular samples on clothing and mobile phones; similar observations were described by Blackman et al. [6]. Lots of DNA could be lost when fingerprints were sampled by ParaDNA collector. So if we plan to use swabs to obtain fingerprint samples for full STR analysis, the ParaDNA guidelines need to be strictly adhered to during sampling (samples are only collected from half of fingerprints) [9].

### Application of the ParaDNA intelligence test to different mock case scenarios

The ParaDNA profiles of items in case scenarios 1 and 2 were shown in Table 2. In the sexual assault scenario, the ParaDNA intelligence test showed that the blood stains on the victim matched the seminal stains in the victim’s bedroom. The collected “suspect” reference buccal sample did not match any samples at the scene. In addition, blood stains recovered from the suspect’s shirt matched the reference buccal sample collected from the victim. The above results confirmed that the “suspect” arrested was not the individual who left evidence at the scene, which was consistent with our simulation. The ParaDNA intelligence test provided fast identification or elimination of suspect/victim profiles and could rapidly link or exclude individuals to the scenes.

In the human trafficking scenario, the ParaDNA intelligence test showed that the reference buccal sample of the suspect matched both the DNA recovered from the cigarette ends found at scene and the DNA recovered from a shirt found in the house. The reference buccal sample of the victim matched the paper cups found at the scene and the blood recovered from pillowcases in the bedroom. The above evidence suggested that the suspect and the victim had both stayed in the house.

All of the results presented in this section were generated within 75 min after the samples were ready for the ParaDNA intelligence test. The two cases were simulated, and the results were consistent with our expectations.

**Table 2.** Detailed ParaDNA profiles of items in case scenario 1 (sexual assault) and case scenario 2 (human trafficking).

Item	D16		D18		TH01		D8		Amelo		D3	
	Allele 1	Allele 2	Allele 1	Allele 2	Allele 1	Allele 2	Allele 1	Allele 2	Allele 1	Allele 2	Allele 1	Allele 2
**Case scenario 1**												
Blood stain on the victim (suspect)	11	13	13	16	6	9	12	13	X	Y	–	–
Seminal stain (suspect)	13	–	13	16	6	9	13	–	X	Y	–	–
Reference buccal sample (unrelated suspect)	**9**	**9**	**12**	**15**	**8**	**9**	**14**	**–**	**X**	**Y**	**15**	**16**
Blood stain on the suspect (victim)	9	11	13	15	7	9	12	16	X	X	15	–
Reference buccal sample (victim)	9	11	13	15	7	9	12	16	X	X	15	–
**Case scenario 2**												
Cigarette ends (suspect)	9	–	17	17	9	–	15	–	X	Y	14	15
Shirt found in house (suspect)	9	13	17	17	9	–	–	–	X	Y	15	–
Reference buccal sample (suspect)	9	13	17	17	9	9	13	15	X	Y	14	15
Blood swabbed from pillowcase (victim)	9	11	13	15	7	9	16	–	X	X	15	15
Paper cups found in house (victim)	11	–	15	–	7	9	12	16	X	X	15	–
Reference buccal sample (victim)	9	11	–	15	7	9	13	–	X	X	15	–

The line in bold indicates a profile of an unrelated reference suspect.

### Cross-validation with other profiling platform

**Figure 4.**
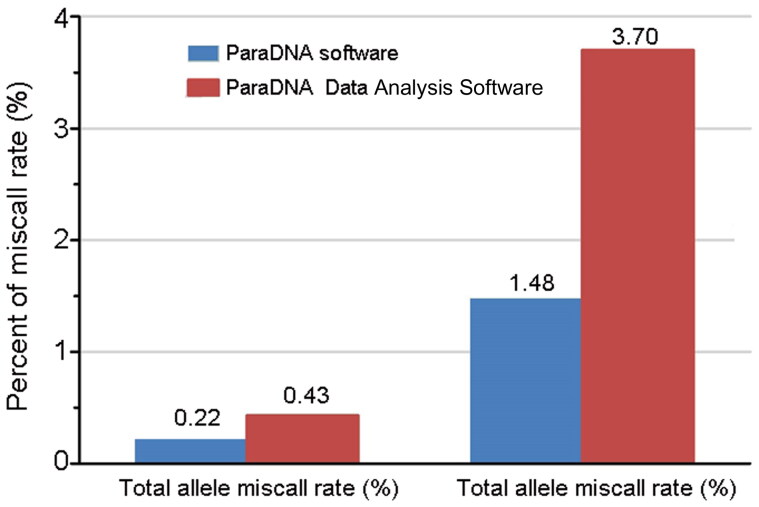
The proportion of alleles and profiles that were different between the ParaDNA intelligence test and the Goldeneye^TM^ DNA ID System 20 A Kit.

To test the accuracy of the profiles generated using the ParaDNA intelligence test, we cross-validated the profiles generated using the ParaDNA intelligence test with those generated using the Goldeneye^TM^ DNA ID System 20 A Kit (an available commercial kit commonly used in forensic investigation) on the CE platform. A group of 135 samples were used for benchmarking the ParaDNA intelligence test against the results generated by the Goldeneye 20 A system. Theoretically, 135 samples should produce 135 kinds of accurate profiles. By comparison, the ParaDNA intelligence test identified a total of 904 alleles, with two alleles that were potentially miscalled (i.e., different from the results of Goldeneye 20 A system) with an allele miscall rate of 0.22% and a profiles miscall rate of 1.48% (Figure 4). After analysis using the ParaDNA Data Analysis Software, a total of 1 157 alleles were detected, with five alleles potentially miscalled, and the miscall rate was 0.43%. Similarly, the profile miscall rate was 3.70% (Figure 4). Among the five potentially miscalled alleles, four were identified from blood on FTA cards. There could be some possible issues with sample preparation or materials of FTA cards. As mentioned above, the ParaDNA Data Analysis Software produces more useful allelic information than the ParaDNA software. Therefore, the risk of miscall could be increased especially with low level of DNA, and it should be more careful when analyzing samples with low amount DNA. Undoubtedly, for samples with high level DNA, results were highly accurate. The results suggested high concordance between the ParaDNA system and the established Goldeneye 20 A system, which was consistent with previous reports [10].

## Conclusion

In this paper, the validation of the ParaDNA intelligence test was assessed in terms of sensitivity, success rate of mock casework samples, mock case scenarios and accuracy. Our findings demonstrate that the ParaDNA^®^ Intelligence System generates useful DNA profiles for samples abundant in DNA, such as blood, saliva and semen. Additionally, for the “cellular samples” containing a low level of DNA, the system could also provide certain STR information for forensic practitioners. The validation studies also indicate that the ParaDNA^®^ Intelligence System is a convenient, fast and robust system that delivers a DNA profile within 75 min, making the system suitable for fast detection to gain rapid investigative leads and intelligent prioritization of samples in the forensic application of human identity testing.

ParaDNA^®^ Intelligence System is also capable of rapid detection in the laboratory or field through the use of portable laboratory or field instruments. ParaDNA intelligence test supports the strategic deployment of cases by classifying biological samples, assisting the screening of objective physical evidence and providing rapid tactical intelligence. ParaDNA^®^ Intelligence System is not a replacement for the current technology or processes but an intelligent rapid biological screening tool, which can save time and improve efficiency for the current DNA identification process.
